# The role of positional changes in optimizing OSA treatment: evidence from DISE

**DOI:** 10.1007/s00405-025-09314-y

**Published:** 2025-03-10

**Authors:** Michaela Mladoňová, Katarína Fedorová, Ondřej Jor, Jana Slonková, Adéla Kondé, Pavel Komínek, Petr Matoušek

**Affiliations:** 1 Department of Otorhinolaryngology and Head and Neck Surgery, University Hospital Ostrava, 17. Listopadu 1790, 70800 Ostrava, Czech Republic; 2 Faculty of Medicine, University of Ostrava, Ostrava, 70800 Czech Republic; 3 Department of Anesthesiology, Resuscitation and Intensive Medicine, University Hospital Ostrava, Ostrava, 70800 Czech Republic; 4 Department of Neurology, University Hospital Ostrava, Ostrava, 70800 Czech Republic; 5 Department of Applied Mathematics, Faculty of Electrical Engineering and Computer Science, VSB-Technical University of Ostrava, Ostrava, 70800 Czech Republic; 6 Department of the Deputy Director for Science, Research and Education, University Hospital Ostrava, Ostrava, 70800 Czech Republic

**Keywords:** Obstructive sleep apnea, Drug-induced sleep endoscopy, Positional therapy, Tongue base obstruction

## Abstract

**Purpose:**

This study aimed to assess the impact of positional changes on upper airway obstruction patterns during drug-induced sleep endoscopy (DISE) in patients with obstructive sleep apnea (OSA) and identify the airway regions most responsive to this change. Special focus was placed on the tongue base, a critical area in OSA pathophysiology.

**Methods:**

This prospective study was conducted from June 2021 to June 2024. DISE was performed in patients with obstructive sleep apnea (OSA) in supine and lateral positions to simulate the effect of positional therapy. Findings were evaluated using the VOTE classification.

**Results:**

The examination was performed on 186 patients, with a median Apnea–Hypopnea Index (AHI) of 19.3. In the supine position, complete obstructions were noted at the soft palate (88.2%), oropharynx (33.3%), tongue base (53.2%), and epiglottis (15.6%). Lateral positioning significantly reduced obstructions, particularly at the tongue base, where obstruction resolved in 94/99 of cases (94.9%). This improvement was significantly more pronounced at the tongue base than at other sites (p < 0.001).

**Conclusion:**

These results suggest that DISE can identify airway regions responsive to positional changes, potentially guiding clinical decisions on positional therapy. The findings show a significant reduction in tongue base obstruction during lateral positioning in DISE. Since tongue base obstruction is a key contributor to airway collapse in OSA, this improvement suggests a practical, non-invasive treatment approach. While these findings highlight an acute association between lateral positioning and reduced obstruction, further studies are needed to evaluate its long-term clinical efficacy.

## Introduction

OSA is a prevalent disorder marked by repeated upper airway collapse during sleep, with the tongue base being a significant site of obstruction in 60–70% of cases [1]. Tongue base obstructions are particularly difficult to manage and often lead to suboptimal treatment outcomes [2, 3].

Several studies confirm that the gold treatment standard, positive airway pressure ventilation (PAP), often fails or requires higher and uncomfortable pressures for the patient to overcome tongue base obstruction [4]. Among other conservative options, the mandibular advancement device is a viable alternative but has relatively low long-term compliance, similar to PAP [5].

Surgical treatment does not guarantee effectiveness unequivocally, and the correct indication is essential. The latest sophisticated and attractive surgical methods, such as transoral robotic surgery or hypoglossal nerve stimulation implants, offer a minimally invasive alternative to traditional approaches with often excellent results [6–8]. However, a significant limitation is the availability of technology and associated costs with a particular risk of disease recurrence [9].

For many years, positional therapy was considered a secondary option due to challenges in evaluating its effectiveness and limited technological solutions [10]. Recent advancements have renewed interest in positional therapy, improving both adherence and patient comfort [11]. Despite these improvements, positional therapy remains underutilized, with indications often based solely on polysomnography findings without considering the specific localization of airway obstruction.

Understanding how body position affects the retromandibular space offers a promising avenue for treating OSA, especially in patients with predominant tongue base obstruction. Lateral sleeping positions have been observed to reduce airway collapse, potentially decreasing OSA severity without requiring invasive procedures or high-pressure PAP therapy [10, 11]. However, there is a lack of standardized approaches for identifying which patients are most likely to benefit from positional treatment, particularly regarding specific obstruction sites.

DISE provides a dynamic, real-time assessment of upper airway collapse patterns, making it a valuable tool for evaluating the impact of positional changes on airway obstruction [3, 12]. Although previous studies have explored positional therapy broadly, few have specifically focused on its effect at individual airway levels, particularly the tongue base [2, 12]. Moreover, evidence is limited regarding the use of DISE to guide individualized treatment strategies for positional therapy. This study aims to address these gaps by investigating the impact of positional changes on upper airway obstruction patterns during DISE, with a focus on the tongue base. We hypothesize that lateral positioning during DISE can help identify airway regions, particularly the tongue base, that are acutely responsive to positional changes. By improving our understanding of the positional responsiveness of upper airway sites, this study seeks to provide a foundation for developing more targeted, non-invasive treatment strategies and enhancing clinical decision-making in OSA management.

## Materials and methods

This prospective study was performed in accordance with the Declaration of Helsinki and the requirements of good clinical practice. It was approved by the local ethics committee. The study was registered in ClinicalTrials under the number NCT02855515. Written informed consent was obtained from each patient before any procedure was initiated.

### Study design and patients

This prospective study was performed at the tertiary referral center from June 2021 to June 2024. Adult patients with OSA diagnosed by overnight polysomnography were consecutively enrolled. Inclusion criteria comprised patients for whom surgical treatment was considered (AHI 5–30 episodes/hour) and those who either did not tolerate PAP or refused PAP therapy (AHI > 15 episodes/hour). Age, gender, BMI, and medication use were not relevant for the inclusion criteria, as patients with varying characteristics were enrolled. Patients were excluded if they had significant comorbidities representing an excessive risk for general anesthesia (decompensation phase)—cardiac, liver, kidney disease, cancer, craniofacial malformations, neurological pathologies, if they were pregnant, or if they disagreed to be included in the study. Patients with suspected but undiagnosed OSA were also excluded.

### Clinical evaluation

Patients were evaluated by collecting a comprehensive history that covered sleep habits and disturbances. As a subjective measure of a patient’s sleepiness, the Epworth sleepiness scale (ESS) was used. Body Mass Index (BMI), age, and gender were recorded. Clinical evaluation included a complete head and neck examination. The upper airways and digestive tract were examined using a flexible video endoscope with a 2.5-mm diameter (Karl Storz, Tuttlingen, Germany). The examination was performed while conscious, and the patient’s position was upright and in a sitting position.

### Drug-induced sleep endoscopy in the supine position

Sleep endoscopy was performed in the operating room with cooperation with the anesthesiologist. The patient was induced to sleep with intravenous propofol (an initial 1 mg/kg bolus, followed by 20–30 mg every 3–5 min). The depth of anesthesia was measured using the bispectral index in the range 50–70. Vital signs were monitored. Sleep endoscopy was performed using a flexible video endoscope with a 2.5-mm diameter (Karl Storz, Tuttlingen, Germany). The examination length was 10–15 min. The results were evaluated using the Kezirian VOTE classification, for which obstruction is assessed in the four localities of the upper airways—the area of the soft palate, the lateral walls of the pharynx and palatal tonsils, the base of the tongue and the epiglottis. In each of these localizations, the degree of obstruction (0—no obstruction; 1—partial obstruction; 2—complete obstruction) and configuration of obstruction (anterior; circular; laterolateral) are evaluated [13]. DISE findings were assessed by a single reviewer.

### Drug-induced sleep endoscopy in the lateral position

**Fig. 1:**
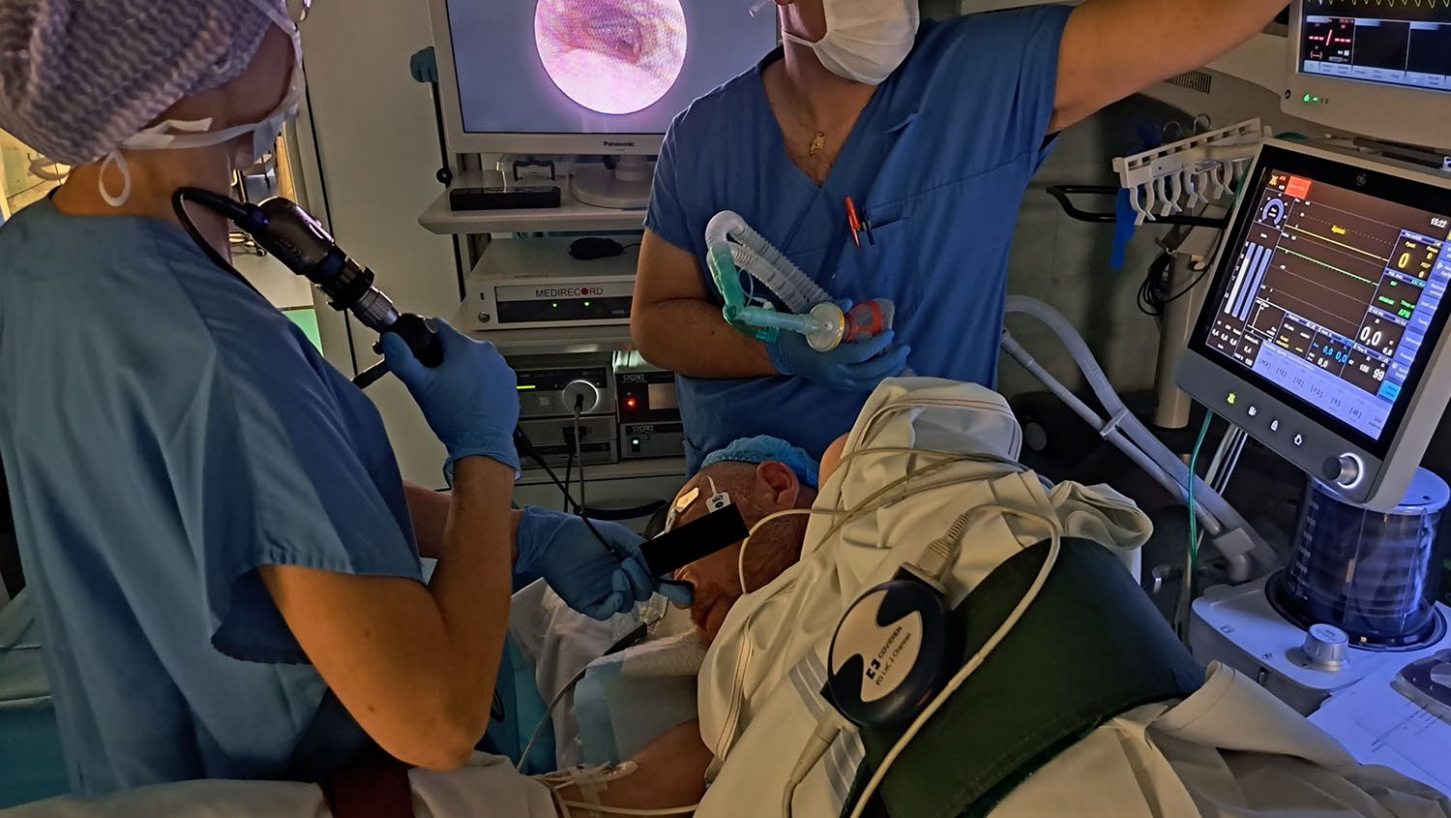
DISE in lateral position; external view

After 10–15 min of the examination, the position was changed from supine to right-side. Subsequently, a flexible endoscope was inserted into the nasopharynx, and it was observed whether there was a change in the condition in individual areas of the upper airways (Fig. 1). The results were evaluated using the Kezirian VOTE classification [13]. The findings were compared with a supine position. It was evaluated how the tongue base area reacts to the change in position against other regions.

### Statistical analysis

Numerical variables were presented as medians and interquartile ranges (IQR). Categorical variables were presented as absolute and relative frequencies (%). The Chi-square test for equality of proportions was used to compare the effects of positional therapy, supplemented by the post-hoc analysis based on Pearson’s residuals. Multiple logistic regression was employed to investigate the potential relationships of selected demographic and anamnestic parameters and their association with the effect of positional therapy. A 100% stacked barplot was used for visualization of the degree of obstruction before and after positional therapy. The significance level was set to 0.05, and all statistical analyses were performed using R software (version 4.4.1).

## Results

One hundred eighty-six patients (39 women, 147 men) aged 30–65 were enrolled in the study. The median AHI was 19.3, and the median BMI was 28.4 kg/m^2^ (Table 1). 57/186 (30.6%) suffered from mild OSA, 98/186 (52.7%) patients had moderate OSA, and 31/186 (16.7%) had severe OSA. During DISE in the supine position, complete soft palate obstruction was observed in 164/186 (88.2%) patients, complete oropharyngeal obstruction in 62/186 (33.3%) patients, complete tongue base obstruction in 99/186 (53.2%) patients, and complete epiglottic obstruction (collapse) in 29/186 (15.6%) patients (Table 2). Single-level obstruction was detected in 9/186 patients (4.8%), and multilevel obstruction (two or more regions) in 177/186 patients (95.2%). Obstruction was observed at two sites in 63/186 patients (33.9%), at three sites in 92/186 patients (49.5%), and at all four locations in 22/186 patients (11.8%).

**Table 1:** Demographical data, entrance limited polygraphy, and ENT examination while conscious (n = 186 patients)

	Median [IQR] or n (%)
Sex, male	147 (79.0)
Age, years	44 [35; 49]
BMI, kg/m^2^	28.4 [26.4; 30.2]
AHI	19.3 [14.0; 27.5]
T90, %	1.20 [0.40; 3.90]
ENT examination while conscious	
Soft palate obstruction	184 (98.9)
Oropharynx obstruction	48 (25.8)
Tongue base obstruction	108 (58.1)
Epiglottic obstruction	5 (2.7)
Mallampati	
I	14 (7.5)
II	62 (33.3)
III	82 (44.1)
IV	28 (15.1)
Friedman	
0	10 (5.4)
1	117 (62.9)
2	37 (19.9)
3	17 (9.1)
4	5 (2.7)

The values represent the median and interquartile range [IQR] or absolute and relative frequencies (%)

**Table 2:** Degree and type of obstruction and analysis of the overall improvement in patients with complete obstruction with lateral position (positional therapy) (n = 186)

	Soft palate	Oropharynx	Tongue Base	Epiglottis	*p*
Obstruction – degree					< 0.001
Complete	164 (88.2)	62 (33.3)	99 (53.3)	29 (15.6)	
Partial	14 (7.5)	69 (37.1)	49 (26.3)	13 (7.0)	
No	8 (4.3)	55 (29.6)	38 (20.4)	144 (77.4)	
Obstruction – type					**–**
Concentric	95 (51.1)	–	–		
Laterolateral	4 (2.2)	130 (69.9)	–	2 (1.1)	
Anteroposterior	79 (42.4)	1 (0.5)	148 (79.6)	40 (21.5)	
No obstruction	8 (4.3)	55 (29.6)	38 (20.4)	144 (77.4)	
Improvement of patients with complete obstruction	82/164 (50.0)	28/62 (45.2)	94/99 (94.9)	19/29 (65.5)	< 0.001^a^

The numbers represent absolute frequencies and relative frequencies (%)

The p-value was obtained using the chi-square test for equality of proportions

^a^Homogenous subgroups (post-hoc analysis): (Tongue Base), (Soft palate, Oropharynx, Epiglottis)

### Effect of lateral position on individual areas

**Fig. 2:**
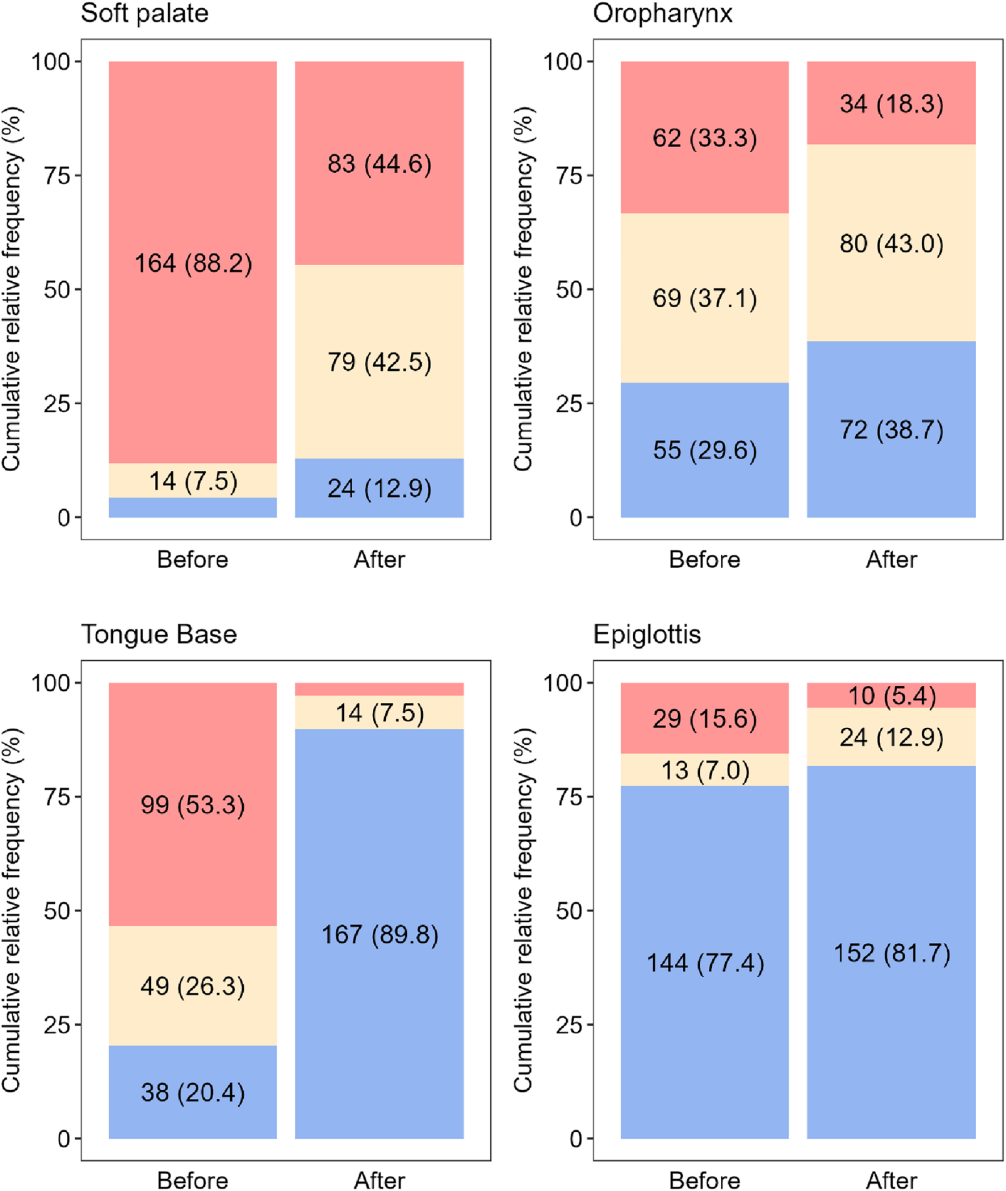
Visualization of the structure of patients based on the degree of obstruction before and after positional therapy for each localization (labels for subcategories with a relative frequency below 5% were omitted for better readability)

The effect of lateral position on the disappearance of complete obstruction in individual areas was analyzed. The lateral position was most effective for complete anteroposterior obstruction of the base of the tongue, where the disappearance of the obstruction was observed in 94/99 (94.9%) cases (Table 2, Fig. 2). The disappearance of the tongue base obstruction was observed significantly more often compared to the other localities of the upper airways.

Two types of obstruction were observed in the soft palate area—concentric and anteroposterior (Table 2). Of the 71 patients with complete anteroposterior obstruction, improvement was seen on repositioning in 35/71 (49.3%) and in 45/90 (50.0%) patients with complete concentric obstruction. The difference between both types of soft palate obstruction in the probability of improvement was not statistically significant (p > 0.999).

The soft palate, oropharynx, and epiglottis areas did not differ significantly from each other and were significantly worse responsive to a change in position (Table 2, Fig. 2).

**Fig. 3:**
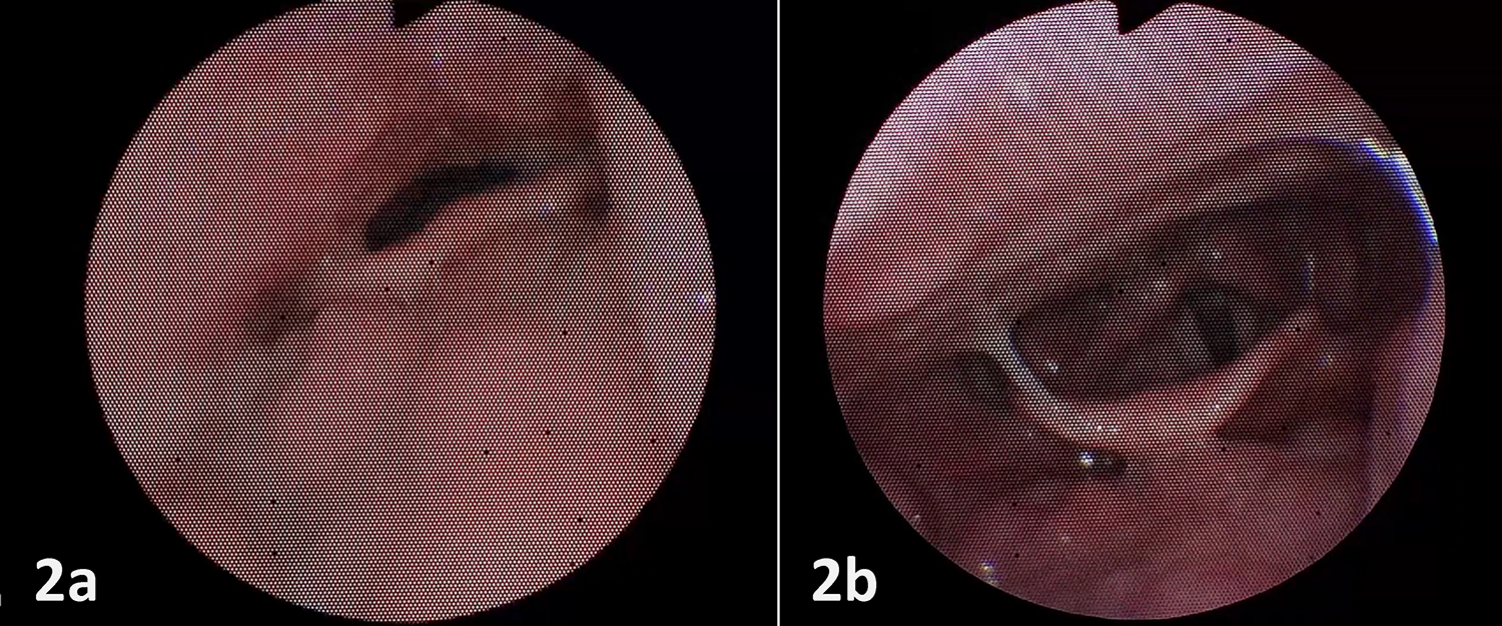
The effect of positional therapy during DISE; 2a—complete tongue base obstruction in supine position; 2b—disappearance of the tongue base obstruction in lateral position; endoscopic view

These findings are supported by the multivariate analysis, which included sex, age, BMI, and AHI. According to the multiple logistic regression model (Table 3), neither sex, age, BMI, or AHI is significantly associated with the probability of improvement. Likewise, the tongue base obstruction showed significantly higher odds of improvement than the selected reference localization (soft palate) and other complete obstruction localizations (Fig. 3).

**Table 3:** Multiple logistic regression model for improvement with positional therapy – age, BMI, AHI, sex, and localization of complete obstruction as covariates (only patients with complete obstruction included)

	OR (95%CI)	*p*
Age (years)	1.01 (0.99; 1.04)	0.290
BMI (kg/m^2^)	0.93 (0.85; 1.00)	0.055
AHI	0.99 (0.98; 1.01)	0.448
Sex		
Female	1.67 (0.87; 3.25)	0.126
Male	Reference	–
Localization of complete obstruction		
Soft palate	Reference	–
Oropharynx	0.94 (0.51; 1.73)	0.852
Tongue Base	18.82 (7.91; 55.79)	< 0.001
Epiglottis	1.88 (0.83; 4.51)	0.140

The values represent odds ratios (OR), 95% confidence intervals (95% CI) and p-values of the Wald test

## Discussion

Positional obstructive sleep apnea (POSA) is a subtype of OSA characterized by the predominance of apnea events occurring when the patient sleeps in a supine position [10]. This condition is influenced by gravitational effects on the upper airway, leading to airway collapse and subsequent breathing disruptions [14, 15]. It occurs in more than half of patients with OSA [16].

Positional therapy is defined as any technique used to prevent the worst position from causing POSA [10, 17]. It has emerged as a viable treatment, demonstrating significant efficacy in reducing the AHI compared to traditional methods. Research indicates that positional therapy can substantially improve AHI, with studies showing reductions from an average of 22.9 to 5.3 events per hour [18].

Positional treatment was not routinely used in clinical practice because it needed a reliable tool for measuring compliance and continued effectiveness [10]. The impossibility of verifying regular use has been a strong argument that significantly limits this treatment method. Therefore, positional treatment is still considered an alternative secondary option [10, 19].

Several tools, devices, and techniques, such as specialized vests and pillows, encourage patients to avoid sleeping on their backs [15, 20]. However, sophisticated electrical sensors with alarms in chest belts have also been newly developed. Thanks to the position sensor, they detect the patient's position during sleep and indicate with vibrations that it is possible to change without fully waking up. The devices not only have an in-built position sensor but can also measure hours of use. These newly developed devices make it possible to break down the previous limits and thus increase the popularity and use of positional therapy [19]. New devices have shown good tolerance and compliance rates, with 90% of patients using the device for over six hours per night [20]. In addition, the use of specialized devices and techniques has improved adherence to positional therapy and made it an attractive alternative [19]. Ramos et al. demonstrated a cost saving by incorporating positional therapy into the treatment algorithm for OSA compared to using PAP alone. The study consisted of 42 patients with OSA, six patients opted for weight management, and one for an oral appliance. Twelve of the remaining 35 patients had POSA and were treated with positional therapy, and 23 were treated with PAP. Positional treatment in the 12 patients with POSA yielded savings of 24% compared to using PAP for all 35 patients [10].

While positional therapy shows promise, it may not be suitable for all patients or universally applicable. The indication is commonly based on polysomnography findings [21]. Polysomnography can identify patients likely to benefit from positional therapy, particularly those with a supine-to-non-supine AHI ratio ≥ 2 [22]. When sleep monitoring evaluates insufficient time spent in the non-supinated position, POSA often remains undetected, and treatment is subsequently not indicated [5, 10].

DISE is currently the primary diagnostic method for identifying sites of obstruction in patients with OSA [3, 23]. DISE helps tailor treatment strategies based on individual anatomical findings by providing dynamic insights into airway obstruction. It is mainly performed before planned surgical treatment [3, 24]. According to recent research and our opinion, DISE also plays a crucial role in selecting patients for positional therapy [2, 12]. Studies indicate that specific maneuvers during DISE enable the selection of patients who may benefit from positional therapy and, at the same time, treatment, thus, can be targeted [25]. However, it has yet to fully clarify how individual areas of the upper airways respond to changes in sleeping position.

In our study, partial improvement (at least in one upper airway location) was observed in all patients in the lateral position. The tongue base showed the highest response to positional changes, with obstruction resolving in up to 94.9% of cases. This improvement was significantly more frequent compared to the soft palate, oropharynx, and epiglottis. According to Victores et al., at least partial resolution of obstruction of the tongue base and collapse of the epiglottis was observed in almost all patients with positional OSA in the lateral sleep position [26]. In our group, improvement in the lateral position was observed in 65.5% of cases of epiglottic collapse. This finding is clinically significant, as several studies have shown that epiglottic collapse is often resistant to PAP therapy, and applying pressure can exacerbate the obstruction [4, 27]. Torre et al. state that PAP does not resolve primary epiglottis collapse, and too high pressures are required to open it [27]. We suggest that positional therapy could serve as a conservative alternative for certain patients with epiglottic collapse. In our study, only 45.2% of oropharyngeal obstructions improved in the lateral position. These findings are consistent with the study by Lan et al., which identified lateral wall collapse of the oropharynx as the sole anatomical predictor of non-positional dependency in OSA patients [28].

The tongue base is one of the most challenging areas to address in OSA management. This complexity arises from its anatomical position and the nature of obstructions [29]. The gold treatment standard, PAP, sometimes fails or requires higher and uncomfortable pressures for the patient to overcome the obstruction [4]. Torre et al. reported that obstructions of the base of the tongue require higher pressure to open the upper airways, with an average pressure of 15.0 hPa [4]. Lai et al. also reported that higher pressure is needed in cases with tongue base obstruction and that this area is essential for correct PAP setting [30]. Excessively high overpressure can burden the patient with treatment-induced central sleep apnea, middle ear ventilation disorders, and other problems that ultimately contribute to inadequate treatment or low compliance [31].

Surgical options (ablation, radiofrequency, or tongue suspension) are also limited. They still present significant challenges and variable complication rates (especially bleeding), with some techniques showing complication rates as high as 42.42% [32].

Various minimally invasive techniques, such as transoral robotic surgery (TORS), have been developed to manage tongue base obstruction. It has emerged as a promising treatment, but the potential for severe complications necessitates careful patient selection and surgical planning. A systematic review indicated a success rate of 69% in reducing the AHI [33]. The hypoglossal nerve stimulation implant's latest sophisticated and attractive surgical method promises a safe and effective treatment, especially for PAP refractory OSA [34]. Although new surgical methods bring better results and fewer side effects, a significant limitation is the availability of technology limited to specialized centers with a particular risk of disease recurrence [9].

Despite advancements in surgical techniques, the inherent difficulties in addressing tongue base obstruction highlight the need for ongoing research.

Positional therapy is offered here as a method that could definitively cure the patient with tongue base obstruction or at least be a suitable additional treatment in the presence of multilevel obstruction. Ravesloot et al. investigated the AHI reduction in patients with multilevel obstruction after uvulopalatopharyngoplasty. It was shown that postoperatively, significantly better results were recorded in the side position, and at the same time, the AHI difference in both positions was considerably higher than before surgery [35].

Our findings demonstrate that positional therapy is highly effective in resolving tongue base obstruction during DISE, likely due to gravitational effects and reduced posterior tongue displacement in the lateral position. Differences in muscle tone and airway collapsibility may also influence the response to positional changes, as suggested by previous studies [14, 28].

Clinically, these results highlight the potential of DISE to support patient stratification for positional therapy. Patients with isolated tongue base obstruction observed during DISE could benefit from positional therapy as a primary or adjunctive treatment, while those with multi-level or non-positional obstruction may require alternative approaches, such as PAP or combined therapies. DISE is a simple and valuable tool for identifying airway regions responsive to positional changes, enabling more tailored treatment strategies [12, 26]. Beyond its diagnostic value, DISE findings can guide conservative treatment decisions by distinguishing patients suitable for positional therapy alone from those needing combination therapy with PAP. Incorporating DISE into individualized treatment protocols offers several advantages: it improves patient selection by identifying those most likely to benefit from positional therapy, supports personalized treatment plans based on specific obstruction patterns, and may reduce PAP dependence. For patient intolerant to PAP, positional therapy informed by DISE could provide an effective, non-invasive alternative, enhancing both adherence and quality of life.

The examination must be conducted properly to ensure accurate results. Beelen et al. reported that partial head rotation or incomplete changes in body position during DISE are inadequate and can underestimate the potential effect of positional therapy in up to one-third of cases [36]. In our study, patients were fully rotated to the side during each assessment. A potential limitation of this procedure is the requirement for additional personnel or the challenges posed by patients with excessive body weight.

The study has several additional limitations. A recent analysis indicates that positional OSA is more prevalent in patients older than 65 years [37]. Since this study includes only patients under 65 years, this should be acknowledged as a limitation, as it may affect the generalizability of the findings to older populations where positional OSA is more common.

Additionally, the reliance on DISE findings as a surrogate for real-world outcomes warrants further discussion. DISE outcomes may not fully correlate with long-term clinical effectiveness in real-world settings. Furthermore, the lack of data on long-term adherence to positional therapy limits the study’s ability to assess sustained benefits over time. Another potential limitation is that DISE findings were evaluated by a single reviewer, which may introduce measurement bias. Lastly, conducting the study at a single tertiary center limits its generalizability, as findings may not translate well to other clinical environments with differing patient populations or healthcare structures. A broader, multi-center approach in future research would enhance the external validity of these findings.

## Conclusion

Patient positioning during DISE provides valuable insights into airway regions responsive to positional changes, potentially guiding clinical decisions on positional therapy. In our study, lateral positioning was highly effective in resolving tongue base obstruction, with a success rate of over 94.9%. This improvement was observed significantly more often at the tongue base compared to other airway regions. Since tongue base obstruction is a major contributor to airway collapse in OSA and can be challenging to manage with surgery or PAP alone, positional therapy offers a practical, non-invasive treatment option. However, as treatment outcomes were not evaluated, these findings indicate an acute association rather than predictive efficacy. Further studies are needed to assess the long-term clinical relevance.

## Data Availability

The data supporting this study are not publicly available due to ethical reasons, but can be obtained from the corresponding author upon reasonable request.
